# Architectural Design of a Blockchain-Enabled, Federated Learning Platform for Algorithmic Fairness in Predictive Health Care: Design Science Study

**DOI:** 10.2196/46547

**Published:** 2023-10-30

**Authors:** Xueping Liang, Juan Zhao, Yan Chen, Eranga Bandara, Sachin Shetty

**Affiliations:** 1 Department of Information Systems and Business Analytics Florida International University Miami, FL United States; 2 American Heart Association Dallas, TX United States; 3 Virginia Modeling, Analysis and Simulation Center Old Dominion University Suffolk, VA United States

**Keywords:** fairness, federated learning, bias, health care, blockchain, software, proof of concept, implementation, privacy

## Abstract

**Background:**

Developing effective and generalizable predictive models is critical for disease prediction and clinical decision-making, often requiring diverse samples to mitigate population bias and address algorithmic fairness. However, a major challenge is to retrieve learning models across multiple institutions without bringing in local biases and inequity, while preserving individual patients’ privacy at each site.

**Objective:**

This study aims to understand the issues of bias and fairness in the machine learning process used in the predictive health care domain. We proposed a software architecture that integrates federated learning and blockchain to improve fairness, while maintaining acceptable prediction accuracy and minimizing overhead costs.

**Methods:**

We improved existing federated learning platforms by integrating blockchain through an iterative design approach. We used the design science research method, which involves 2 design cycles (federated learning for bias mitigation and decentralized architecture). The design involves a bias-mitigation process within the blockchain-empowered federated learning framework based on a novel architecture. Under this architecture, multiple medical institutions can jointly train predictive models using their privacy-protected data effectively and efficiently and ultimately achieve fairness in decision-making in the health care domain.

**Results:**

We designed and implemented our solution using the Aplos smart contract, microservices, Rahasak blockchain, and Apache Cassandra–based distributed storage. By conducting 20,000 local model training iterations and 1000 federated model training iterations across 5 simulated medical centers as peers in the Rahasak blockchain network, we demonstrated how our solution with an improved fairness mechanism can enhance the accuracy of predictive diagnosis.

**Conclusions:**

Our study identified the technical challenges of prediction biases faced by existing predictive models in the health care domain. To overcome these challenges, we presented an innovative design solution using federated learning and blockchain, along with the adoption of a unique distributed architecture for a fairness-aware system. We have illustrated how this design can address privacy, security, prediction accuracy, and scalability challenges, ultimately improving fairness and equity in the predictive health care domain.

## Introduction

The ability to identify patients at a high risk for life-threatening diseases is essential for precision medicine. The integration of artificial intelligence (AI) and digital health data such as electronic health records (EHRs) can improve precision medicine by enabling better diagnosis and prediction [1]. Previous work has demonstrated that using machine learning (ML) with EHR data can improve the prediction accuracy of adverse outcomes such as cardiovascular disease [2] and enable the early detection of symptoms of COVID-19 [3]. However, as precision medicine has evolved, many research projects have been conducted at local levels or by using local EHR data, which can introduce bias owing to underrepresented population samples and siloed data sources. Although solutions have been proposed [4] to train and build global models with data from various local institutions, several critical issues hinder widespread adoption, such as the high cost associated with data transmission and storage as well as the high risk in security and privacy [5]. In addition, unclear data ownership and restrictions on data sharing further impede progress in this area [6].

Federated learning (FL) is an ML paradigm in which multiple collaborative sites only share locally trained ML models while keeping all the training data private [7]. Studies have shown that FL-trained models can achieve performance levels comparable with those trained using centrally hosted data sets and are superior to those trained with single-institution data alone [8,9]. Therefore, developing health AI technologies using FL is essential and in high demand in the field of medicine [10]. One such example is the privacy-preserving federated ML (FML) projects supported by the European Union Innovative Medicines Initiatives. However, most existing FL systems rely on centralized coordinators, which are vulnerable to security attacks and privacy concerns because of the possible single point of failure.

To fill these gaps, this study aims to detect the bias in health care data, improve the fairness of predictive models using FL, and enhance trust and fairness through blockchain-assisted FL. We propose a blockchain-empowered, decentralized FL platform that improves fairness in predictive models in the health care domain while preserving privacy. We adopted a blockchain platform to establish ML models with the existing data on its off-chain storage. Specifically, our design follows a 2-cycle research method. By embedding fairness metrics in the federated setting with a blockchain consensus process, our design improves the overall fairness in the global model and provides feedback to update the local training models. We implemented the design and prototype using the Aplos smart contract, microservices, Rahasak blockchain, and Apache Cassandra–based distributed storage. In a pilot study [11] that involved 5 simulated medical centers as peers in the Rahasak blockchain network, we demonstrated how our design improved the accuracy of a predictive model using 20,000 local model training iterations and 1000 federated model training iterations. Our evaluation results show that the proposed design provides accurate predictions while providing fairness with an acceptable overhead. Our innovative design contributes to health care equity and quality of care by providing accurate and fair clinical decisions [12].

## Methods

### Ethical Considerations

We did not collect any human-related information or any survey from any uses. Data used in this paper is generated by algorithms to test the system performance.

### Overview of Algorithmic Fairness and FL

The definition of fairness in ML is 2-fold: statistical notions of fairness and individual notions of fairness [13]. Statistical definitions of fairness refer to a guarantee of parity across protected demographic groups based on statistical measures, whereas individual definitions of fairness require equal treatment for individuals with similar features [13,14]. Algorithmic fairness has been viewed as a sociotechnical phenomenon in recent literature [15], and there are mutual influences between the technical and social structures. The use of AI algorithms is sophisticated, and there are no standards or guidelines to guarantee that the algorithms are designed with fairness [16] or lead to fair outcomes. Despite the existence of unfairness in AI algorithms, people can be incentivized to use an algorithm when they could modify their forecasts [17]. Human control of algorithms or human behaviors as the input of algorithms could play an essential role in algorithm fairness. Moreover, although research has made progress on methods for measuring and addressing algorithmic fairness [18], such as IBM AI Fairness 360 toolkit [19], most existing studies have focused on a centralized ML setting, which is not directly applicable to the FL setting. As FL avoids full access to the raw training data set, finding methods to detect and address bias without directly examining sensitive information is an open challenge.

Fairness is also a major concern in health care. Specifically, the problems of equity in access to care and the type of care received are predominant concerns related to health care quality [20,21]. As a solution, ML is increasingly being used in health care to address these concerns. However, research on algorithmic fairness in precision medicine is scant. Current work on fairness may provide a general approach in a general context [22]. Such a general approach may not solve all fairness problems for precision medicine because precision medicine involves multiple unique types of data that can cause different types of bias in ML models. In short, there is a need to measure, audit, and mitigate the bias in FL specific to precision medicine applications while protecting privacy during the data collection, processing, and evaluation phases. Several studies show that aggregating statistics may lead to the possibility of identifying individuals [23]. In particular, in FL, the capabilities of sharing models and data among distributed agents can lead to data leakage via reverse engineering and aggregation of models. There are growing concerns for data being reidentified and used without patients’ consent or knowledge [24]. Prior research suggests a general “debiasing” method by removing redundant encoding related to sensitive human attributes for prediction-focused ML applications [25]. However, this method was tested only in the credit-lending context and may not be ideal for decision-making in a health care context. Another method relates to the human-centric, fairness-aware automated decision-making (ADM) framework [26] that emphasizes the holistic involvement of human decision makers in each step of ADM. This method is unrealistic in health care, given the complexity of medical decision-making and privacy challenges.

### Blockchain and FL Integration in Health Care

In an FL system in health care, a central coordinator coordinates the learning process and aggregates the parameters from local ML models trained on local participant data sets. A centralized coordinator is vulnerable to various security attacks and privacy breaches because it runs the risk of a single point of failure. Moreover, in FL, malicious actors can exploit the distributed model training process. They can fool the algorithm by sharing fake data, incorrect gradient, or model parameters. In addition, FL does not address issues that are inherent to learning on medical data. Health data are subject to biases, for example, a group of populations over- or underrepresented in the training data and a large number of missing values. The distributed FL mechanism makes it challenging to identify sources of bias. Predictive algorithms trained on such data may also amplify the bias and yield decisions skewed toward a certain group of patient populations, thus inadvertently introducing unfairness in decision-making [27]. Such unfairness would worsen the disparities in health care and harm health equity [28]. Hence, we propose the use of blockchain technology in FL to address the privacy challenge. Blockchain technology provides transparent operations [29] and accountability in a decentralized architecture, while maintaining an acceptable overhead and balanced trade-off between algorithm performance and fairness.

### Design Science Research Methodology

**Figure 1:**
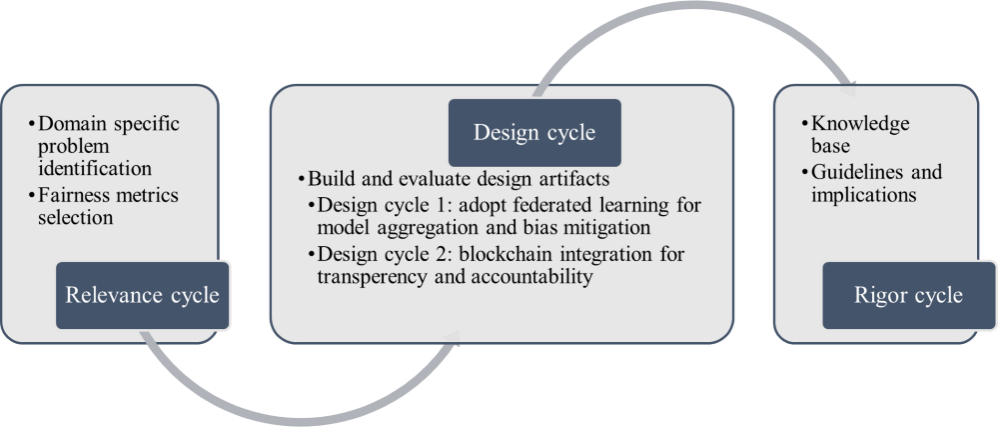
Design science research methodology (adapted from the study by Hevner [30]).

We follow the 3-cycle view of design science research and presented the tasks for each cycle in Figure 1 [30]. In the “Relevance Cycle” section, we describe the objectives of our fairness-aware FL platform and list the design science activities that bridge blockchain and FL to improve fairness. The design cycle iterates between building and evaluating the design artifacts, where we have 2 design cycles. The first design cycle involved adopting FL for fairness improvement in disease prediction. The second design cycle is blockchain integration to enhance the fairness of the FL process for disease prediction. The rigor cycle presents the resources, technology, and expertise available to establish the research project by connecting the design activities with the knowledge base of fairness in health, FL, and blockchain from scientific foundations and implications. We discuss the relevance cycle and the artifacts building in this section and the implementation and evaluation of the 2 design cycles in the following 2 sections.

### Relevance Cycle

#### Problem Identification

The focus of this study was to understand the issues of bias and fairness in the ML process for prediction in the health care domain. Following the design science research framework proposed in the study by Hevner et al [32], this study aims to understand the issues of bias and fairness metrics in the health care domain and use a software architecture that integrates FL with blockchain to improve fairness with acceptable prediction accuracy and overhead. In doing so, we identified 3 main objectives for this study:

- Objective 1 was to understand and detect the bias in health care data prepared for building predictive models. We proposed objective metrics for fairness so that health care organizations can objectively measure fairness in the design process using fair algorithms and detect biases and unfairness with such objective metrics.
- Objective 2 was to mitigate bias and improve the fairness of predictive models using FL. FL provides opportunities to explore the adoption of various fairness metrics suited for the distributed learning process and overall learning outcomes for predictive models.
- Objective 3 was to develop blockchain-assisted FL for fairness and trustworthiness. We adopted blockchain to assist the FL process to improve the resilience of the architecture by decentralizing the data flow during the model training process. Moreover, the communication architecture using blockchain will ensure accountability and transparency during the model aggregation process and mitigate biases by executing smart contracts and achieving consensus from all participating nodes. Blockchain-assisted FL will also ease the concern of data sharing for health care organizations owing to privacy restrictions in health care.

#### Fairness Metrics

Considering the health care context and objective of the predictive models in medical decision-making, we focused on the fairness of prediction performance. Fairness relates to biases. Bias refers to the disparity observed in both the underlying data and prediction model outcomes trained with the data. We defined disparity as “discrepancies in measures of interest unexplained by clinical need,” in line with the definition of the Institute of Medicine [33]. We consider a model fair if its prediction errors are similar between the privileged and unprivileged groups. In contrast, an algorithm is unfair if its decisions are skewed toward a particular group of the population without being explained by clinical needs [18,33]. On the basis of the definition of fairness [34], we used 2 metrics to assess fairness—equal opportunity difference (EOD) and disparate impact (DI). Textbox 1 shows the terminology used to define the fairness metrics in this study.

**Definition of the terminology [35].**

- Protected attribute: a protected attribute divides sample data into groups that should have parity in their outcomes. In this study, race and gender were 2 protected attributes that we investigated.
- Privileged group: a privileged group was defined as a group of people whose protected attributes have privileged values. For example, White and male groups were the privileged group compared with Black and female groups.
- Label: the outcome label for each individual; 1 indicates a diagnosis (case); 0 means normal control.
- Favorable label: a favorable label is one whose value corresponds to an outcome that benefits the recipient. Positive disease prediction was the favorable label because high-risk patients can be identified early and treated to reduce their risk of adverse outcomes.

Previous studies adopted EOD and DI as fairness metrics [27,36] for individual fairness and group fairness. We chose EOD and DI as the primary metrics of fairness because they were suggested in multiple studies related to bias assessment [18,19,27]. In addition, true positive rates and positive prediction rates are important concerns for fairness in clinical prediction models. We define EOD and DI as follows:

1. EOD measures the difference in true positive rates between the privileged and unprivileged groups. Mathematically, the EOD is defined as follows:



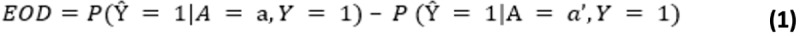



where Ŷ is the predicted label, A is the protected attribute, a is the privileged value (ie, White or men), a′ is the unprivileged value, and Y is the actual label.

An EOD value of 0 indicates fairness if both groups have equal true positive rates, which implies that the probability of an individual with a certain predictive outcome should be the same for Black and White and male and female.

2. DI measures the ratio of predicted favorable label percentage between the groups, defined as follows:



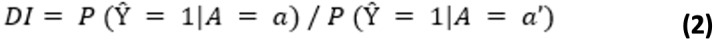



where Ŷ, A, a, and a′ have the same meaning as defined in equation 1. A DI value of 1 indicates fairness if the predicted favorable outcome percentage is the same for both privileged and unprivileged groups. The idea behind DI is that all people should have an equivalent opportunity to obtain a favorable prediction regardless of race and gender.

In the design cycles, we used both local fairness and global fairness to deal with the algorithmic fairness of the proposed design. Local fairness refers to the fairness measure in each local data training process. Each FL node will self-monitor its local state containing the fairness measurement and dynamically adjust the ratio of each feature of the measurement. Both EOD and DI are calculated interactively using feedback from the global training model. On average, global fairness should have similar weights based on the nearness of the predefined level [18]. The blockchain node will monitor the global state containing the fairness measurement and dynamically adjust the ratio of each group. The global EOD and DI will be shared with all participants so that local models can be iteratively improved based on input from the global states. Both local and global fairness metrics will be monitored and recorded in the blockchain for accountability and transparency purposes.

### Design Cycle: Build and Evaluate

#### Design Cycle 1: Adopt FL for Bias Mitigation in Disease Prediction

**Figure 2:**
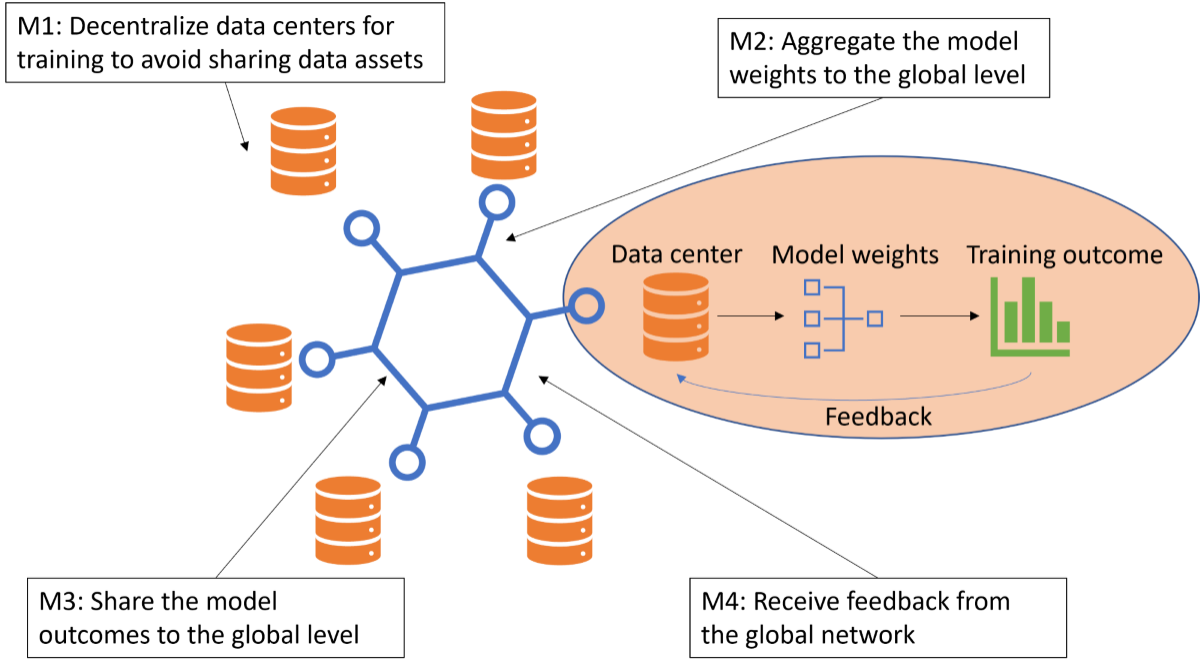
Adopt federated learning for disease prediction.

Design cycle 1 was conducted to adopt FL for bias mitigation in disease prediction. Bias mitigation, or debiasing, attempts to improve the fairness metrics by modifying the training data distribution, the learning algorithm, and the predictions. In this design cycle, we used both local fairness and global fairness to deal with the algorithmic fairness of the proposed design. Local fairness refers to the fairness measure in each local data training process. Each FL node will self-monitor its local state containing the fairness measurement and dynamically adjust the ratio of each feature of the measurement. Both fairness metrics (EOD and DI) were interactively calculated using feedback from the global training model. The global EOD and DI will be shared with all participants so that local models can be iteratively improved based on input from the global states. Both the local and global fairness metrics will be monitored and recorded. We implemented 4 modifications (M1 to M4) in the disease prediction algorithm, as shown in Figure 2, to achieve bias mitigation.

In M1, we used decentralized data processing to perform bias mitigation by retrieving local fairness measurements instead of sharing data among participating institutions. During the model training process, we adopted 2 types of debiasing methods: removing protected attributes from the feature set and resampling to balance the group distribution of the training data across the protected attributes. Both protected attributes and data imbalance are the primary causes of bias. As race and gender were protected attributes, we excluded them from comparison when training the models. Previous studies have shown that removing the race or gender attribute from the prediction model can reduce bias through a mechanism called fairness through unawareness [27,37]. Consequently, we compared the models trained with and without the protected attributes.

In M2, we aggregated the ML model parameters at the global level using the calculated training outcomes. For ML models, bias is most likely caused by either of the following two imbalanced cases: (1) training data in each group have an imbalanced sample size. (2) Class distributions are not the same across all groups. The resampling approach aims to mitigate the bias caused by these 2 imbalanced cases. We applied two resampling methods adapted from the study by Afrose et al [38]: (1) resampling by group size, which oversampled the minority group (smaller sample size) to match the size of the majority group, and (2) resampling by proportion, which resampled only positive samples in the group with a lower ratio of positive class to balance ratios between groups. Resampling by group size was adopted in the study by Afrose et al [38] where there was a sample enrichment process to incrementally enrich a specific subgroup so that a set of candidate models were generated to achieve an optimal model.

**Figure 3:**
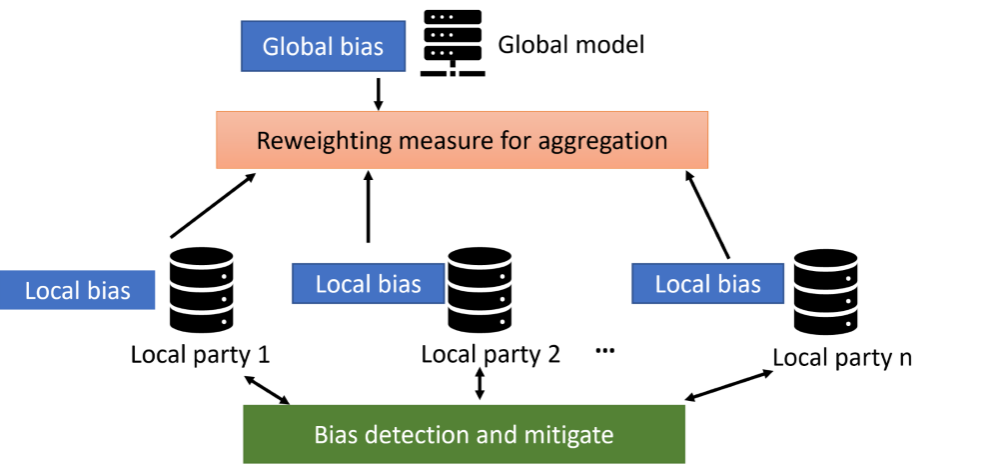
A hierarchical framework to detect and address local and global bias under federated learning.

After M2 aggregates the local model to the global level, each participant shares the model outcomes at the global level in M3 and receives feedback from the aggregated global model in M4. Participants in M3 shared the model data instead of the original data set, preserving the privacy of individual participants. In M4, participants received feedback automatically from the global network supported by the collaborative network and adjusted the local model parameters. Figure 3 shows a hierarchical audit framework to detect and address local and global bias under the FL architecture and the processes of M3 and M4, where the local parties share their local bias with the global model while reweighing measures for aggregation. The global model calculates the global model bias as feedback to each local party to improve their models and initiate the local reweighting process. The reweighting process will be iterated for several rounds until a satisfying threshold has been met.

#### Design Cycle 2: Integrate Blockchain to Enhance Fairness and Trustworthiness

Design cycle 2 involved blockchain integration to enhance the trustworthiness and fairness of the FL process. We designed a blockchain-assisted FL platform to enhance the security and reliability of the FL process. The challenge of this cycle lies in the network design of the FL process on top of the blockchain network and the enhanced measurements to further reduce and mitigate biases to achieve fairness in both local and global models. To address the bias challenge, this study uses the blockchain network to track the data flow and provide feedback to each participating institute for further fine-tuning of parameters for fairness features and the protection of data privacy of distributed nodes. We achieved this with updated modifications (M1 to M4) by removing the central server. Moreover, we achieved real-time and transparent weight adjustment, real-time model outcome adjustment, and peer-to-peer feedback for fairness measurement.

Overall, we propose 3 algorithms to instantiate the blockchain-enabled FL process. In M1, we adopted an incremental learning technique to train the models continuously by multiple peers in the blockchain network. Each peer in the blockchain adopts debiasing methods in their local models. Once a peer generates a model, it can be aggregated incrementally by other peers in M2. Each blockchain node stores the information to be shared in M3. The ledger provides a method to audit the system. This design improves the transparency of the feedback process in M4. To provide training model provenance, a model card framework is adopted [39] for ML model use and ethics-informed evaluations, which are essential operations requiring accountability and transparency. The model card object contains model information, such as participating clients, generators of local models, and aggregators, serving as a traceable record of model development and protection against adversarial ML attacks.

## Results

### System Implementation

**Figure 4:**
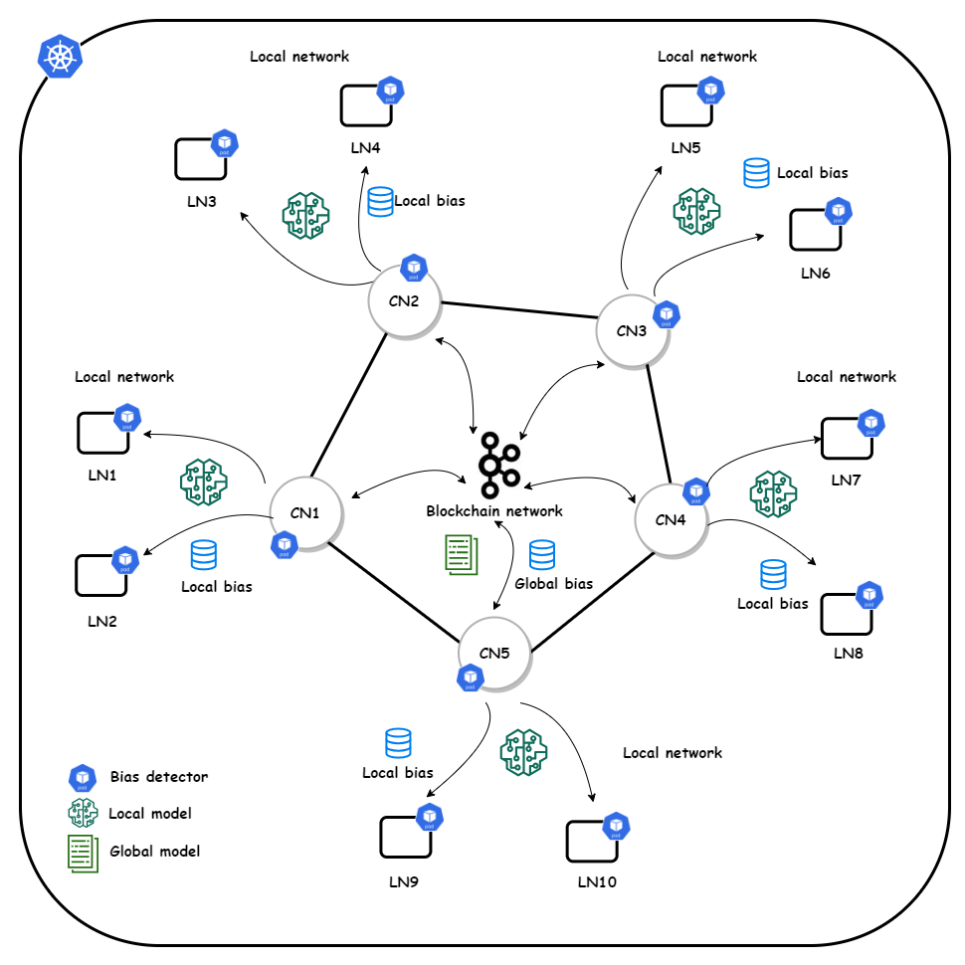
System overview of the layered architecture with collaborative node (CN) and learning node (LN) network.

In the implementation of the blockchain-assisted FL platform, we incorporated an incremental learning process to continuously train the models by blockchain peers (Figure 4). The Rahasak blockchain was adopted [40], with Aplos smart contracts [41] providing a customized smart contract interface. Rahasak is a permissioned blockchain platform in which participating institutions can register and enroll their identities through the membership management service. The platform has been designed using a microservices architecture [42]. In our implementation, we have 2 types of nodes: collaborative nodes, representing collaborative nodes in the blockchain network, and learning nodes, representing learning nodes for the FL process. Each blockchain node in a collaborative nodes comes with 2 microservices: FML service and storage service. The FML service handles FML functions using the Pytorch and Pysyft libraries. The storage service handles the off-chain data storage implemented with Apache Cassandra–based [43] distributed storage. To bootstrap the learning process in individual institutions, we implement algorithm 1 to initialize the training pipeline, assuming a scenario where blockchain nodes are deployed in 3 institutions, institutions A, B, and C (Textbox 2). Blockchain is configured to store the data related to ML models. Each institution has its off-chain storage, which stores local patient data. The ML models are published in the blockchain ledger, with consensus achieved using Apache Kafka [44].

**Algorithm 1: training pipeline initialization.**

- Input: Blockchain nodes information, machine learning model training parameters
- Output: Blockchain genesis block, updated training parameters
- Results
  1. The system initializes with 1 blockchain node chosen by the round robin scheduler with the node information and current training model parameters.
  2. The chosen blockchain node extracts information attributes from the training model parameters and executes the block creation process.
  3. The chosen blockchain node broadcasts the genesis block to other peers in the network and the system choose the next available blockchain node to continue the learning process.
- Return
  1. The results are returned.

We used the Lokka service to generate new blocks, including the genesis block with incremental learning flow [45] and the following blocks containing model parameters. In the new block generation process, we implemented a federated consensus with 3 Lokka services. Blocks 1, 2, and 3 were sequentially generated by Lokka A, B, and C, respectively. This block approval process is repeatedly performed via federated consensus implemented by Lokka services to generate future blocks. We implemented an incremental learning flow that defined the order of the training process. Assume that the incremental learning flow among the 3 institutions is A to B to C. Peer A will produce an initial model to be incrementally trained by peer B and then peer C. Once peer A publishes the genesis block containing model parameters and incremental flow to the entire network, other peers take the block via a distributed cache service in the Rahasak-ML training module and start to retain models based on local data sets.

In the implementation of each incremental learning process, peer A generates the learning model based on the model parameters in the genesis block. The original model was not published, but the hash and uniform resource identifier of the built model were produced as a transaction. Peer B fetches the URI of the model and launches the local training process with off-chain storage. Similarly, this training model will be saved on peer B’s off-chain storage, and peer B will publish the hash and URI of the model as a transaction. Next, peer C repeats the training process. After 3 institutions (or most of the institutions) successfully complete the model training, the Lokka service recognizes a finalized model and generates a new block containing details with the finalized model parameters. Multiple transactions produced by each peer will be included in the new block. This new block is broadcasted to other peers for validation. The peers validate the learning process with the transactions in the block. Once the finalized model has been fetched, it can be shared via smart contracts for prediction.

### System Evaluation Outcomes

#### Overview

As a use case of the platform, we discuss how to explore the integration of FL and blockchain into the health care domain. The blockchain network was deployed in 5 hospitals in a simulated environment, where each had its own data set. We used a data set about inflammations of the bladder to predict acute inflammations of the bladder [46]. A logistic regression algorithm was used to build models for each peer. The evaluation of the platform focuses on model accuracy, performance in terms of blockchain scalability, and overall overhead during model calculations.

#### Descriptive Assessment of Fairness

We used decentralized data processing to perform bias mitigation by retrieving local fairness measurements instead of sharing data among participating institutions. During the model training process, we adopted 2 types of debiasing methods: removing protected attributes from the feature set and resampling to balance the group distribution of the training data across the protected attributes. Both protected attributes and data imbalances were the primary causes of bias.

#### Training and Validation Loss for Local Model Accuracy

**Figure 5:**
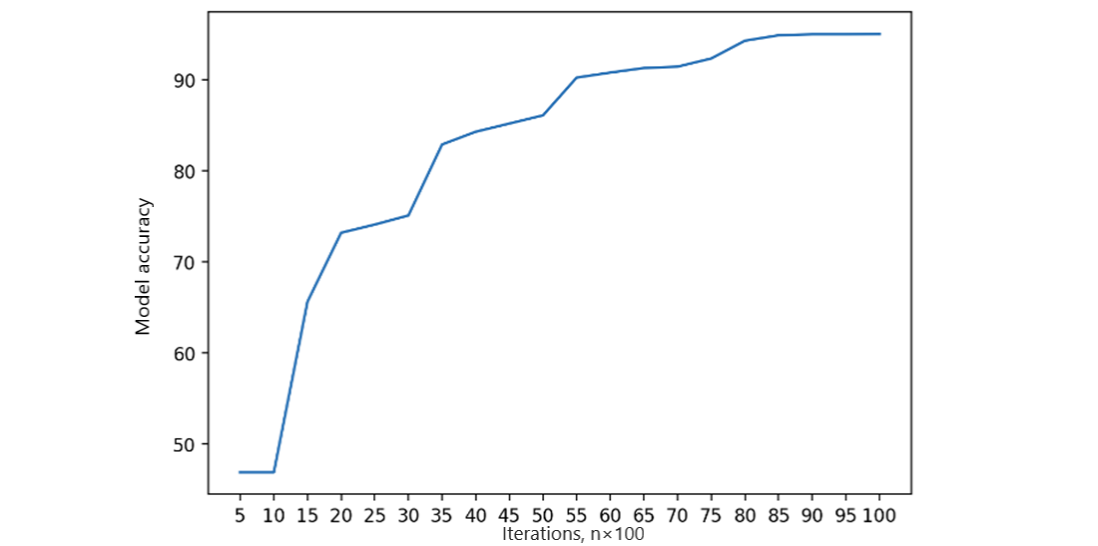
Single peer local model accuracy.

**Figure 6:**
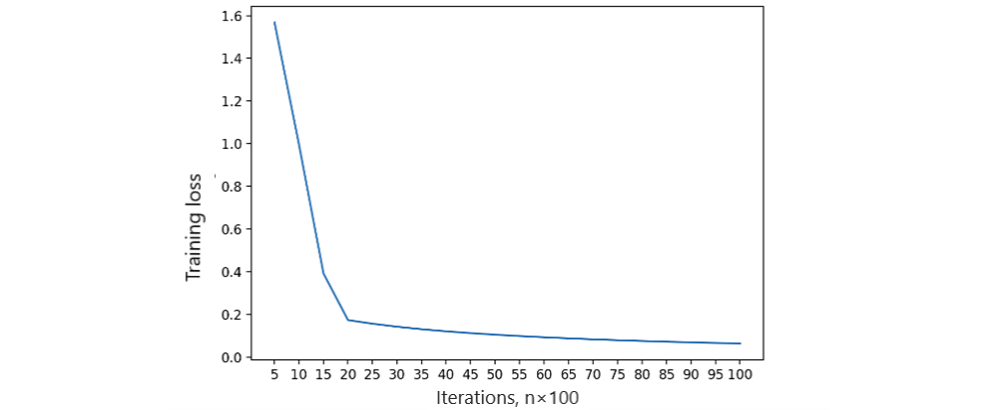
Single peer training loss.

**Figure 7:**
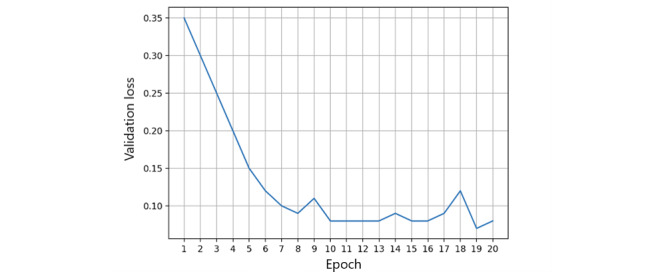
Single peer validation loss.

Local fairness metrics are calculated as the first step of the FL process. Each FL node will self-monitor its local state containing the fairness measurement and dynamically adjust the ratio of each feature of the measurement. The local training process is performed with 20,000 iterations to capture local model accuracy, as well as training and validation loss on a single peer [47]. We measured the accuracy of the local model using the area under the receiver operating characteristic curve (Figure 5). As the number of iterations increases, the accuracy of the local model reaches a steady threshold, indicating that model stability is achieved. Simultaneously, we captured both the training loss and validation loss in Figures 6 and 7 in a single peer, and the results show that the validation loss reaches an acceptable level after 1000 iterations.

#### Federated Model Accuracy

**Figure 8:**
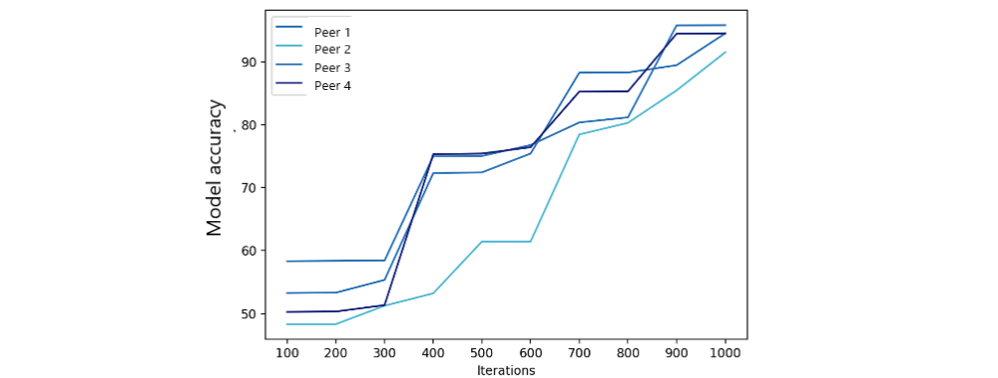
Federated model accuracy.

**Figure 9:**
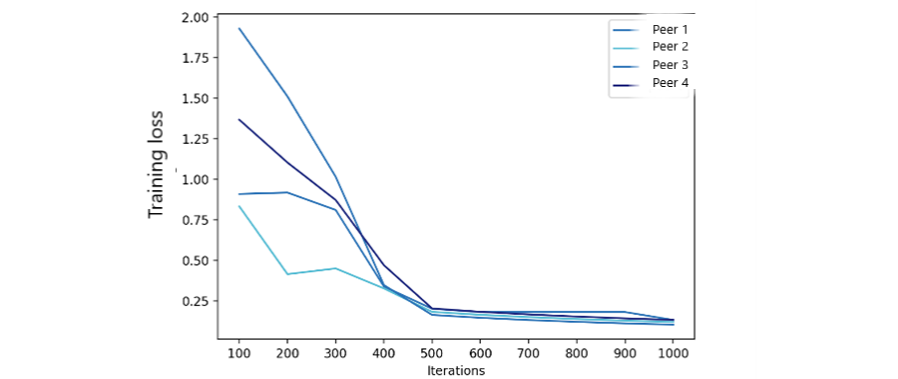
Federated model training loss.

In this evaluation, we used 1000 iterations to measure the model accuracy and training loss of the FML model with different numbers of peers. The resulting accuracy (Figure 8) indicates that as the number of peers increases, the accuracy of the federated model is improved. Similarly, the federated model training loss (Figure 9) is also improved significantly after 500 iterations. The number of peers in the federated training process does not play a significant role because the main modification to the FL process is related to fairness metrics instead of ML parameters.

#### Performance of Blockchain Scalability

**Figure 10:**
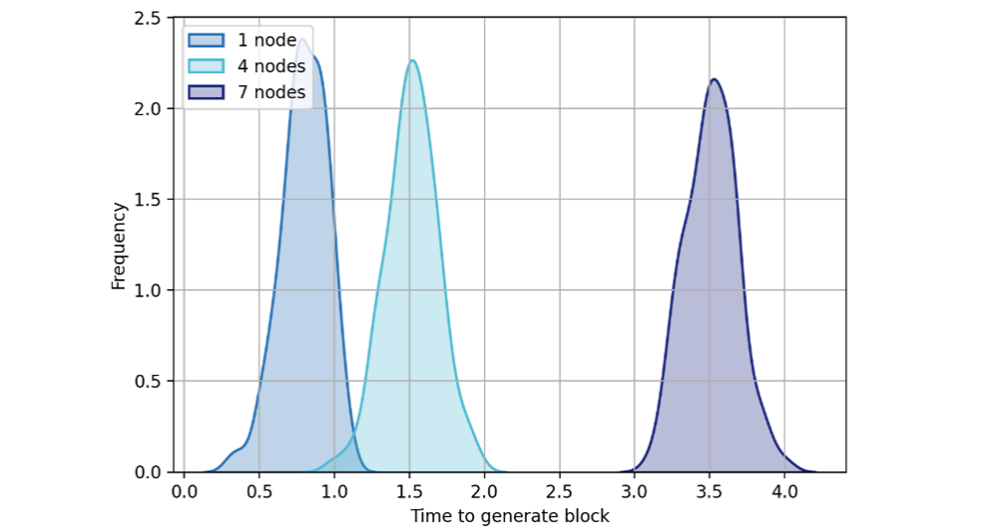
New block generation time.

In the local block generation and consensus phase, blocks will be generated in a preset threshold. The average time required to generate a block depends on the steps required in the consensus process: leader election, peer broadcast, block generation, and transaction validation. Multiple transactions will be included in 1 block, which will greatly improve the performance in terms of scalability. In this evaluation, we simulated the consensus process using 1, 4, and 7 nodes (Figure 10). As new peers join, the time required to generate a new block increases because of the new block calculation and verification. A larger number of peers will consume more time for new block calculation and verification, but the overall performance of the network is improved.

#### Local Model–Building Time and Search Time

**Figure 11:**
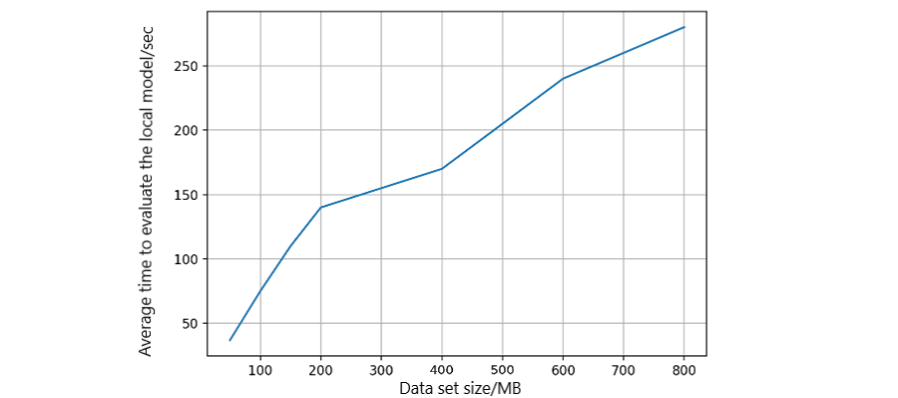
Model-building time in each peer.

**Figure 12:**
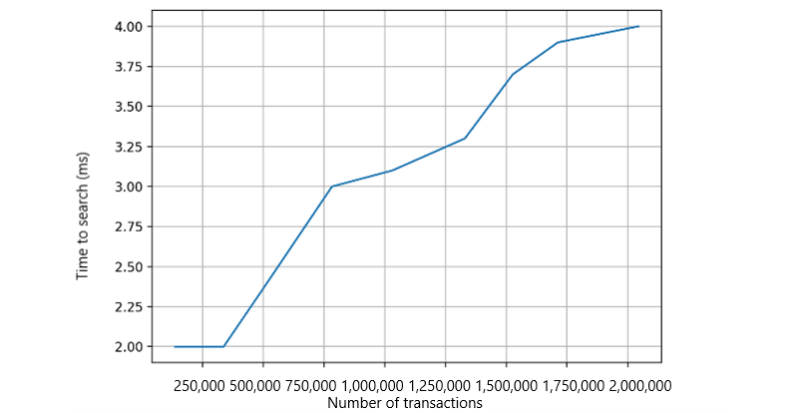
Transaction search in each peer.

In this evaluation, we measured the average model-building time relative to the size of the data set. The ML models are built using a logistic regression algorithm. We adjusted the data sizes in each simulation (Figure 11). Overall, the local model–building time increases linearly and shows the scalability of the platform with varying volumes of data sets. The health record data are stored off-chain in the Cassandra-based Elassandra storage, which allows for transaction search operations. We used elastic search-based APIs to simulate the transaction search operations (Figure 12). When each peer performs transaction searches, the time taken to search against the number of records in the storage increases. The overall time cost is in milliseconds, which is acceptable in ML models.

## Discussion

### Principal Findings

We designed and implemented a bias-mitigation process within the blockchain-empowered FL framework. First, we propose a design of an FL platform that can mitigate bias and thus improve fairness in decision-making in distributed medical institutions without sharing raw health data. Such a design can incentivize collaboration among health care institutions and ease their concerns regarding data leakage and privacy risks in a centralized setting. Second, we integrate blockchain into the FL framework to provide an accountable and transparent bias-mitigation process. Meanwhile, the integration of blockchain and FL enables institutions to share FL models without a centralized coordinator and removes a single point of failure. The implementation of the system demonstrated that the proposed design is a feasible solution for addressing fairness in a decentralized environment. Performance evaluation indicates that the overhead brought about by blockchain integration is acceptable, considering the achieved capabilities.

Our work makes several contributions to the fields of research and practice related to FL for disease prediction and clinical decision-making. To the research field, our study first contributes to the research on FL and blockchain by designing and implementing the blockchain-empowered FL framework that can improve the fairness of decision-making in the health care domain. The framework combines the advantages of FL learning in distributed settings and blockchain in terms of privacy and trustworthiness preservation. Second, it contributes to algorithmic fairness research by implementing a bias-mitigation process through which both the global and local FL learning models can incrementally or continuously improve fairness using the feedback of the fairness metrics adopted. Third, it contributes to design science research by demonstrating how design science research guides analytic research in general and health care analytics in particular. Our work shows that adopting design science for analytic research can help ensure the design rigor of analytic artifacts in a specific domain with system requirements from the user perspective.

Our work has practical implications. It provides a solution to primary stakeholders such as patients and providers who are concerned about fairness and disparity in health care. For patients, our design accounts for biases in training data to avoid the over- or underrepresentation of certain patient populations and hence eases patients’ fairness concerns in ML-aided clinical decision-making. For providers, our design protects the privacy of data for local institutions that are subject to strict data-sharing restrictions under security and privacy regulations. This can motivate collaboration among providers to build more accurate global models to improve the fairness, precision, and quality of clinical decision-making.

For developers, our work provides the blockchain-empowered FL framework, its prototype, and the prototype evaluation. Developers can build upon our work to have a full implementation of the framework to generate learning models with fairness for specific disease diagnoses and clinical decisions. They can also extend our work to explore other designs that combine the advantages of FL and blockchain. In pursuing such designs, they may consider several trade-offs based on our design. First is the trade-off between prediction accuracy and fairness. To achieve fairness, there are metrics to adopt and parameters to adjust, so this will inherently affect the prediction accuracy. Second, there is a trade-off between the prediction accuracy and cost of accuracy. The computational performance during the prediction is largely dependent on the architecture design and number of nodes in the blockchain. To balance the desired accuracy and number of participating health care institutions, developers need to conduct precise modeling and simulation before real-world production. The third trade-off is the fairness and transparency requirement, which enhances model trust and promotes market adoption of innovative architectural design. Overall, human trust should be at the forefront of algorithm development, so that trust in ML models can be understood and promoted.

As for physicians, the proposed platform provides opportunities for incentive design and can help mitigate human bias and structural inequalities that could affect diagnosis and treatment use [48]. Our blockchain-empowered FL platform allows each participant to share their data, thus promoting the fairness of the collaboration. Following our design, an incentive model can be built. The model can consider the contribution of each participant and distribute rewards accordingly, based on the topological relationships between the participants to further develop value models in the process of revenue distribution [49]. This proposed platform can be combined with signature techniques to maintain fair incentives for physicians, such as the Boneh-Goh-Nissim cryptosystem and Shamir’s secret sharing for data obliviousness security and fault tolerance [50]. Moreover, each IT component should not only have an independent fiduciary responsibility to each hospital for the standardization, organization, maintenance, aggregation, and release of data but also be enabled to respond to the needs of collaboration among physicians as a whole [51]. A better understanding of both the cultural and political significance of IT implementation, specifically the algorithm design and new technology adoption, quality of care delivery, and effectiveness, can be incorporated [52].

### Limitations

Blockchain and FL are both new technologies that have not yet been fully developed in the health care domain [53] or framed by government rules and regulations [54]. There are some technical limitations of our prototype, especially related to the health care domain, which are highlighted as follows: in the health care domain, ML models could differ in terms of formats and parameters based on different data sets. Generalized and standardized mechanisms for institutional collaboration are required to address this limitation. Sharing ML models can lead to unintended intellectual information disclosure if the deployment of the system is not done correctly. Using thorough planning and negotiation between institutions [55] can address this limitation. The incentive mechanism for institutional collaboration could also be explored to address this limitation in future work. Full participation from all stakeholders [56] is essential for promoting the adoption of this innovative architectural design to achieve algorithmic fairness. Meanwhile, the sequential nature of FL may limit the efficiency of the learning process. However, in hospital settings, disease prediction is not required to be performed in real time, and the number of nodes is not large. It is acceptable to have delays in the learning process.

### Comparison With Prior Work

FL is an innovative technology in the ML field that addresses health care issues. Previous research provided benchmark data [57] to provide a performance assessment and guidance on privacy-preserving aspects [58] for FML in medical research, including mobile health [59]. Prior research has investigated the combination of FL and blockchain technologies to address unfairness in FL. A weighted data sampler algorithm was developed to enhance fairness in a COVID-19 X-ray detection use case [60], which provides accountability. However, this method does not preserve privacy. When it comes to privacy challenges in FL, research efforts usually focus on statistical inference by combining multiple datasets from different sources. These efforts use methods such as the statistical estimator, risk utility [61], and binary hypothesis testing [62], which are successfully developed in many scenarios with radiation and partitioned data sets [63]. We need models that can configure an appropriate set of attributes or the optimal combination of attributes to identify individuals such as name, address, and telephone number. Existing privacy-preserving applications use decentralized learning mechanisms [64] but face the issue of identity leakage due to the sharing of data and models between distributed nodes. There are growing concerns for data being reidentified and used without patients’ consent or knowledge [24]. Prior research suggests a general “debiasing” method by removing redundant encoding that is related to sensitive human attributes for prediction-focused ML applications [25]. However, this method was tested only in the credit-lending context and may not be ideal for decision-making in a health care context. Another method relates to the human-centric, fairness-aware ADM framework [26] that emphasizes the holistic involvement of human decision makers in each step of ADM. The method is unrealistic in health care, given the complexity of medical decision-making and privacy challenges.

Our proposed model enables institutes to configure an appropriate set of attributes or the optimal combination of attributes to identify individuals such as name, address, and telephone number. Overall, our blockchain technology–enabled FL platform provides transparent operations and accountability on a decentralized architecture while maintaining an acceptable overhead and balanced trade-off between algorithmic fairness and algorithm performance.

### Conclusions and Future Directions

A major goal of precision medicine is the fast and reliable detection of patients with severe and heterogeneous illnesses. However, data from multiple health care providers are heterogeneous with varying characteristics and behaviors, resulting in unfair and inaccurate predictions. Hence, bias needs to be detected and mitigated in different cycles of data from the origin to collection and processing. We designed the mechanism of bias mitigation within the blockchain-empowered FL framework based on a novel architecture design that enables multiple medical institutions to jointly train predictive models using their privacy-protected data effectively and efficiently, ultimately achieving fairness in decision-making in the health care domain. The proposed framework functions in a 2-stage process, namely the learning process and the sharing or coordination process. The learning process was initiated at each participating institute, where data were locally collected, stored, and used for training local ML models. The sharing or coordination process is initiated when the participating institute joins the collaborative network with permission from the blockchain membership management service. The sharing or coordination process is mainly responsible for bias reduction and mitigation, based on the adopted fairness metrics.

This novel architectural design helps to understand and detect the bias in health care data prepared for building predictive models. It mitigates bias and improves the fairness of predictive models using FL. To do so, it develops blockchain-assisted FL for fairness and trustworthiness and to improve the resilience of the architecture by decentralizing the data flow during the model training process. Our system evaluation shows that the proposed design provides accurate prediction while providing fairness with an acceptable overhead. Hence, our design can help improve health care equity and the quality of care by offering accurate and fair clinical decisions. Local hospitals can benefit from the FL process in which what is learned in peer hospitals is integrated. This enables hospital systems to benefit from one another without sharing patient data.

Future work can extend this framework and create a collaboration model that incorporates the incentive mechanism to promote participation and collaboration among relevant health care institutions. The incentive model can evaluate the contribution of each participant and distribute the rewards accordingly. Future work can extend this framework by embedding different fairness metrics and evaluating the fairness outcomes. Future studies can further improve our work by testing it using various medical data sets. Moreover, research has attempted to integrate new learning methods, such as swarm learning, with new technologies, such as edge computing and fog computing, for ML models while maintaining confidentiality without the need for a central coordinator. Future work can build on our work by applying new learning methods.

## Abbreviations

ADM: automated decision-making
AI: artificial intelligence
DI: disparate impact
EHR: electronic health record
EOD: equal opportunity difference
FL: federated learning
FML: federated machine learning
ML: machine learning

## Data Availability

The data sets generated during and/or analyzed during this study are available from the corresponding author on reasonable request.
